# Esketamine use is associated with shortened postoperative hospital stay in patients after knee arthroscopic surgery: a propensity score–matched cohort study

**DOI:** 10.1186/s12871-023-02376-7

**Published:** 2024-01-17

**Authors:** Jing Liu, Hong Han, Shangze Yang, Xiaoxuan Zhan, Bingbing Cao, Yue Peng

**Affiliations:** 1 Department of Anesthesiology, the Eighth Affiliated Hospital, Sun Yat-sen University, No.3025 Shennan Road, Futian District, Shenzhen City, Guangdong Province China; 2 Department of Anesthesiology, Guangdong Provincial People’s Hospital (Guangdong Academy of Medical Sciences), Southern Medical University, Guangzhou, China

**Keywords:** Knee arthroscopy, Esketamine, Postoperative length of stay

## Abstract

**Background:**

Previous studies have examined anesthetics to improve postoperative prognosis after knee arthroscopic surgery. However, it is currently unknown whether perioperative anesthetics can influence postoperative hospital stay. We investigated the impact of esketamine after knee arthroscopic surgery on post-operative length of stay, fever and surgical site infection.

**Methods:**

This study included 455 patients who underwent knee surgery between January2020 and August 2021at a tertiary hospital in China. Patient characteristics, preoperative laboratory values, intra-operative anesthetic data, and postoperative outcomes were collected. Univariate and multivariate logistic regression analyses with or without propensity score matching were performed to identify factors related to post-operative discharge within 3 days(PD3).

**Results:**

A total of 297 cases met our inclusion criteria. The mean age of patients was 42 ± 14 years, mean body mass index, 24.1 ± 3.5 kg/m^2^, 157(53%) patients were male. Meniscus-related procedures accounted for the most part of all the procedures with a percentage of 40.4%, followed by combined procedures of 35.4%. After we adjusted for demographic and intraoperative characteristics with propensity score matching, esketamine use was significantly associated with PD3 with the highest odds ratio of 2.28 (95% confidence interval (CI): 1.18–4.41, *p* = 0.014).

**Conclusion:**

Esketamine use was associated with PD3 in patients underwent knee arthroscopic surgery. The findings of this study will be useful to anesthesiologists in making informed decisions regarding the choice of anesthetics for knee joint diseases.

**Trial registration:**

This study was approved by the Ethics Committee (Approval No.:2023-041-01) of the Eighth Affiliated Hospital, Sun Yat-sen University and retrospectively registered.

## Key message

- This study explored the relationship between perioperative anesthetics and postoperative discharge within 3 days(PD3) in patients underwent knee arthroscopic surgery.
- Esketamine use is an independent factor influencing PD3 in patients underwent knee arthroscopic surgery.

## Introduction

Knee arthroscopy is a novel and minimally invasive surgical technique that has gained significant attention in the field of orthopedic surgery [1]. It involves the use of an arthroscope to visualize and treat internal knee joint diseases [2]. Over the past two decades, the number of knee arthroscopy surgeries, especially outpatient surgeries, has grown exponentially due to the numerous advantages it offers over traditional knee surgery [1]. Compared to traditional knee surgery, knee arthroscopy has the benefits of smaller incisions, less bleeding, fewer postoperative complications, and faster recovery time [3].

Numerous clinical trials have provided evidence for the potential of perioperative anesthetics in improving postoperative prognosis for patients underwent knee arthroscopic surgery [4–6].For example, in patients undergoing anterior/posterior cruciate ligament reconstruction, intravenous dexmedetomidine in combination with intraarticular bupivacaine could significantly lower the need for analgesic [7]. Esketamine, a commonly used perioperative analgesic, has been widely applied in various surgical procedures [8–11]. A meta-analysis based on randomized controlled trials (RCTs) has indicated that esketamine can effectively alleviate postoperative pain, reduce analgesic consumption, and not significantly increase the incidence of nausea and vomiting in knee arthroscopic surgery [12]. Additionally, in elderly individuals undergoing hip arthroplasty, esketamine has been demonstrated to alleviate short-term postoperative anxiety and depression, while also providing relief from postoperative pain and stress response [13]. However, the potential effects of perioperative anesthetics on length of hospitalization following knee arthroscopy remain scarcely investigated. Minimizing hospital stays is a major advantage of minimally invasive procedures, leading to reduced costs and expedited recovery. Therefore, the aim of this study is to investigate whether perioperative anesthetics can be identified as influential factors in post-operative hospital stay for patients undergoing knee arthroscopic surgery and whether they can improve patient prognosis based on a retrospective analysis of data.

## Methods

As this is a retrospective study, informed consent from patient was waived as approved by the Ethics Committee (Approval No.: 2023-041-01) of the Eighth Affiliated Hospital of Sun Yat-sen University, we reviewed the medical records of 455 patients who underwent knee surgery between January 2020 and August 2021 and all methods were performed following the Declaration of Helsinki. In order to determine an optimal sample size that is cost-effective, we plan to incorporate 3 to 4 independent predictors into a multivariate logistic regression model. The predicted probability of the primary dichotomous outcome - patients being discharged within 3 postoperative days - is estimated as 25%. With a significance level of 0.05 and a sample of 297 patients, we can achieve sufficient statistical power of over 80% to detect meaningful effects.

### Data collection

Since postoperative hospital stay was not normally distributed, the upper quartile range of which was used to define shortened postoperative hospital stay. The upper quartile range was 3 days, and patients were dichotomized into two outcome groups based on whether they were discharged within three days postoperatively (PD3). The primary outcome measure was the incidence of PD3 and secondary outcomes included postoperative readmission, postoperative fever and surgical site infection. The patient’s demographic records we obtained included age, sex, preoperative hypertension, diabetes, preoperative hemoglobin (Hb), aspartate aminotransferase (AST), ASA classification, and surgery history. The intraoperative data recorded for each patient included type of anesthesia, type of surgery, duration of operation, intraoperative use of corresponding anesthetics and intraoperative fluid infusion. The identification of one certain anesthetic use was based on the anesthetic record, including intraoperative use and postoperative patient controlled intravenous analgesics. Esketamine was administered either intraoperatively or postoperatively depending on the anesthesiologist’s preference, with total doses ranging from 0.25 to 1.50 mg/kg.

### Data processing

We recorded the data in specialized form on an Excel spreadsheet and imported them into SPSS 26 statistical software and R package version 3.0.2 for analysis. Normality of distribution was checked graphically and by using the Shapiro–Wilk test. Continuous data were summarized and reported as the mean(SD) or medians (interquartile range) and were compared using Student’s independent t-test or the Mann-Whitney-U test according to the type of distribution. Categorical variables were presented as the total number and percentage and compared using the chi-squared or Fisher’s exact test depending on the number of events as appropriate.

In our study, we conducted a thorough examination of multicollinearity, during which we discovered that the Variable Inflation Factor (VIF) for WBC exceeded the threshold of 10. Consequently, WBC was excluded and was not incorporated into either the univariate or multivariate analyses. In order to ensure the robustness of our analysis, we maintained the independence of observations by rigorously adhering to a well-defined data collection and sampling protocol. This approach was designed to eliminate any interdependencies among observations.

To assess the robustness of our findings regarding PD3, we conducted a sensitivity test using propensity score matching. After adjusting for variables, logistic regression was employed to choose a 1-to-1 matching score. “Nearest neighbor matching” was employed as the propensity score matching method, implemented through the “matchit” function. To present balance post-matching, we utilized the “CreateTableOne” function. Factors with univariate analysis results showing a p-value less than 0.1 were included in the multivariate logistic regression analysis. A P value of < 0.05 was deemed statistically significant.

## Results

**Fig. 1:**
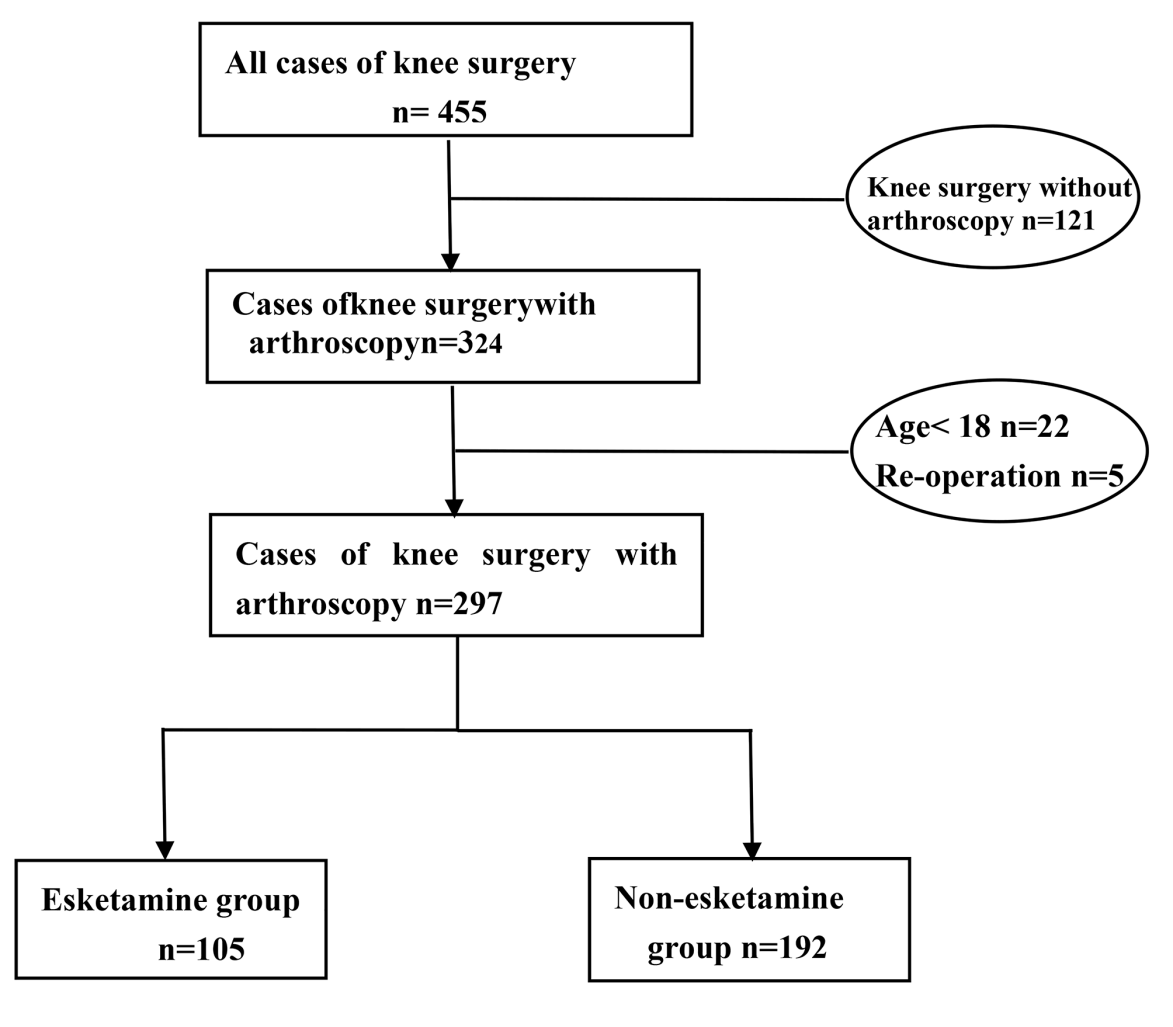
Flow diagram of cases undergoing knee arthroscopic surgery

131 of the 455 patients who underwent knee surgery were not operated with arthroscopy and 22 for whom age was less than 18 were excluded. Ten repeated cases (five male and five female) were detected, five of whom underwent different side of knee surgeries at different hospitalization times were included while five were excluded because of readmission and operation on the same side, leaving data for 297 patients available for analysis (Fig. 1). The mean age of the patients was 42 ± 14 years (mean ± SD), including 157 males and 140 females. Of the 297 included patients, 80(26.9%) were discharged within 3 days (Table 1).

The factors associated with PD3 after knee arthroscopic surgery before propensity score matching are presented in Table 2. Surgery duration and esketamine use were significantly associated with PD3 as assessed by univariate logistic regression. Dexamethasone use were trending significant (*P* < 0.1) for PD3. All factors with *P* < 0.1 in the univariate analyses were entered into the multivariate logistic regression analysis. Esketamine use and surgery duration remained significantly associated with PD3. Of note, no influence on PD3 was found with respect to dexamethasone use (*P* = 0.142).

Table 3 compares the baseline characteristics of patients who received esketamine (Esketamine group, n = 105) and those who did not (Non-esketamine group, n = 192). Age was significantly lower in the esketamine group than in the non-esketamine group (*P* = 0.005), patients with hypertension rate was 4.8% in the esketamine group and 13.0% in the non-esketamine group (*P* = 0.040), while both remained unsignificant after propensity score matching.

The factors associated with PD3 after knee arthroscopic surgery after propensity score matching are presented in Table 4. Surgery duration, and esketamine use were significantly associated with PD3 as assessed by univariate logistic regression and multivariate logistic regression.

Table 5 compares the adverse events of patients who received esketamine(esketaminegroup, n = 105) and those who did not(non-esketamine group, n = 105) after propensity score matching. The rate of postoperative fever, surgical site infection and re-operation was not statistically different between the esketamine group and the non-esketamine group.

**Table 1:** Patient characteristics

Variables	N = 297
Age	42 ± 14
Sex(male)	157(52.9%))
BMI	24.1 ± 3.5
ASA status I II III	187(63.0%) 106(35.7%) 4(1.3%)
Diabetes	13(4.4%)
Hypertension	30(10.1%)
WBC	6.1(5.1–7.5)
AST	19(16–25)
Hemoglobin	137 ± 31.9
Glucose	5.1 ± 0.9
Creatinine	67 ± 16.3
General anesthesia	93(31.3%)
Surgery duration	82(60–124)
Emergency surgery	14(4.7%)
Surgical procedure	
Meniscus	120(40.4%)
Joint	28(9.4%)
Ligament	34(11.4%)
Combined	105(35.4%)
Other	12(4.0%)
Dexmedetomidine	247(83.2%)
Propofol	136(45.8%)
Sevoflurane	37(12.5%)
Esketamine	105(35.4%)
Dexamethasone	129(43.4%)
Tramadol	29(9.8%)
Colloids PD3	14(4.7%) 80(26.9%)

ASA, American Society of Anesthesiologists; AST, Aspartate Aminotransferase; PD3, postoperative discharge with 3 days.

**Table 2:** Univariate and multivariate analysis of patients who were discharged postoperatively within 3 days before propensity score matching

Variables		Univariate analysis		Multivariate analysis	
		OR (95% CI)	P-value	OR (95% CI)	P-value
Age		1.01(0.99–1.03)	0.420		
Sex(male)		0.95(0.57–1.59)	0.852		
BMI		0.97(0.90–1.04)	0.397		
ASA status					
I		0.89(0.52–1.51)	0.659		
II		1.04(0.61–1.78)	0.880		
III		1 (ref)			
Diabetes		0.57(0.18–1.81)	0.344		
Hypertension		1.02(0.43–2.38)	0.972		
AST		1.01(0.99–1.01)	0.274		
Hemoglobin		1.01(0.99–1.02)	0.212		
Glucose		0.99(0.76–1.32)	0.979		
Creatinine		0.99(0.98–1.01)	0.596		
Generalanesthesia		1.18(0.67–2.07)	0.563		
Surgery duration		0.99(0.98–0.99)	< 0.001	0.99(0.98–0.99)	< 0.001
Emergency surgery		2.28(0.50-10.43)	0.287		
Surgical procedure					
Meniscus		0.67(0.40–1.13)	0.131		
Joint		0.91(0.39–2.17)	0.838		
Ligament		0.87(0.40–1.91)	0.730		
Combined		1.39(0.80–2.41)	0.242		
Other		4.22(0.54–33.21)	0.172		
Dexmedetomidine		0.94(0.47–1.89)	0.870		
Propofol		1.48(0.88–2.50)	0.140		
Sevoflurane		0.85(0.40–1.82)	0.682		
Esketamine		2.11(1.18–3.77)	0.012	2.14(1.17–3.90)	0.013
Dexamethasone		0.62(0.36–1.05)	0.076	1.52(0.87–2.65)	0.142
Tramadol		1.18(0.48–2.87)	0.721		
Colloids		2.28(0.50-10.43)	0.287		

**Table 3:** Characteristics of patients who received esketamine and those who did not. Data are presented as the mean (SD) or n (proportion)

Parameters	Before propensity score matching			After 1-to-1 propensity score matching		
	Esketamine (n = 105)	Non-esketamine(n = 192)	P-value	Esketamine (n = 105)	Non-esketamine(n = 105)	P-value
Age	39 ± 13.1	44 ± 14.0	0.005	39 ± 13.1	40 ± 11.9	0.908
Sex(male)	59 (56.2%)	98 (51.0%)	0.466	59 (56.2%)	59 (56.2%)	1.000
BMI	24.2 ± 3.65	24.3 ± 3.50	0.693	24.2 ± 3.65	23.7 ± 3.59	0.331
ASA status			0.150			0.234
I	73 (69.5%)	114 (59.4%)		73 (69.5%)	68 (64.8%)	
II	30 (28.6%)	76 (39.6%)		30 (28.6%)	37 (35.2%)	
III	2 ( 1.9%)	2 ( 1.0%)		2 ( 1.9%)	0 ( 0.0%)	
Diabetes	3 ( 2.9%)	10 ( 5.2%)	0.516	3 ( 2.9%)	2 ( 1.9%)	1.000
Hypertension	5 (4.8%)	25 (13.0%)	0.040	5 ( 4.8%)	5 ( 4.8%)	1.000
WBC	6.52 ± 1.66	6.33 ± 1.87	0.380	6.52 ± 1.66	6.34 ± 1.81	0.452
AST	21.6 ± 10.3	22.7 ± 13.2	0.471	21.6 ± 10.3	23.1 ± 10.8	0.299
Hemoglobin	136 ± 17.3	135 ± 16.7	0.808	136 ± 17.3	136 ± 17.5	0.855
Glucose	5.1 ± 0.83	5.1 ± 0.98	0.918	5.1 ± 0.83	5.0 ± 0.73	0.092
Creatinine	67.5 ± 15.0	67.3 ± 16.9	0.886	67.5 ± 15.0	68.1 ± 14.4	0.798
General anesthesia	30 (28.6%)	63 (32.8%)	0.534	30 (28.6%)	31 (29.5%)	1.000
Colloids	2 ( 1.9%)	12 ( 6.2%)	0.161	2 ( 1.9%)	9 ( 8.6%)	0.063
Surgery duration	98 ± 49.0	94 ± 52.0	0.563	98 ± 49.0	98 ± 56.7	0.938
Emergencyoperation	4 ( 3.8%)	10 ( 5.2%)	0.797	4 ( 3.8%)	5 ( 4.8%)	1.000
Surgical types						
Meniscus	44 (41.9%)	76 (39.6%)	0.790	44 (41.9%)	41 (39.0%)	0.779
Joint	11 (10.5%)	17 ( 8.9%)	0.803	11 (10.5%)	9 ( 8.6%)	0.814
Ligament	12 (11.4%)	22 (11.5%)	1.000	12 (11.4%)	13 (12.4%)	1.000
Combined	34 (32.4%)	71 (37.0%)	0.506	34 (32.4%)	36 (34.3%)	0.884
Other	5 ( 4.8%)	7 ( 3.6%)	0.874	5 ( 4.8%)	6 ( 5.7%)	1.000
Dexmedetomidine	87 (82.9%)	160 (83.3%)	1.000	87 (82.9%)	90 (85.7%)	0.705
Propofol	52 (49.5%)	84 (43.8%)	0.405	52 (49.5%)	45 (42.9%)	0.406
Sevoflurane	16 (15.2%)	21 (10.9%)	0.374	16 (15.2%)	15 (14.3%)	1.000
Dexamethasone	38 (36.2%)	91 (47.4%)	0.082	38 (36.2%)	38 (36.2%)	1.000
Tramadol	12 (11.4%)	17 ( 8.9%)	0.610	12 (11.4%)	7 ( 6.7%)	0.336

**Table 4:** Univariate and multivariate analysis of patients who were discharged postoperatively within 3 days after propensity score matching

Variables	Univariate analysis		Multivariate analysis	
	OR (95% CI)	P-value	OR (95% CI)	P-value
Age	1.01(0.99–1.04)	0.406		
Sex(male)	1.27(0.68–2.36)	0.456		
BMI	0.95(0.87–1.04)	0.259		
ASA status				
I	0.82(0.42–1.60)	0.558		
II	1.15(0.59–2.26)	0.677		
III	1 (ref)			
Diabetes	1.40(0.15–12.76)	0.768		
Hypertension	1.41(0.29–6.83)	0.673		
AST	0.99(0.96–1.02)	0.549		
Hemoglobin	0.99(0.98–1.01)	0.543		
Glucose	0.94(0.63–1.41)	0.765		
Creatinine	0.99(0.97–1.01)	0.380		
General anesthesia	0.96(0.49–1.90)	0.913		
Surgery duration	0.99(0.98-1.00)	0.002	0.99(0.98-1.00)	0.002
Emergency surgery	-	-		
Surgical procedure				
Meniscus	0.66(0.35–1.22)	0.184		
Joint	0.79(0.29–2.17)	0.645		
Ligament	1.44(0.51–4.05)	0.488		
Combined	1.26(0.64–2.46)	0.503		
Other	3.63(0.45–29.04)	0.224		
Dexmedetomidine	0.59(0.23–1.53)	0.285		
Propofol	1.35(0.72–2.52)	0.352		
Sevoflurane	0.82(0.35–1.91)	0.647		
Esketamine	2.26(1.19–4.30)	0.013	2.28(1.18–4.41)	0.014
Dexamethasone	1.67(0.85–3.29)	0.138		
Tramadol	1.33(0.42–4.20)	0.627		
Colloids	3.63(0.45–29.04)	0.224		

**Table 5:** Adverse events

	Postoperative fever N(%)	Postoperative surgical site infection N(%)	Readmission N(%)
Group Esketamine(n = 105)	1(1.0%)	2(1.9%)	2(1.9%)
Group non-Esketamine (n = 105)	0(0.0%)	1(1.0%)	3(2.9%)
P value	1.000	0.993	1.000

Data are presented as numbers(percentage).

## Discussion

The duration of hospitalization is a pivotal metric that clinicians and patients employ to assess the surgical prognosis, while simultaneously constituting a vital component of curtailing hospitalization expenses. In the context of knee arthroscopic surgery, elements that may impact the duration of hospital stay encompass patients’ characteristics, the nature of the surgical procedure and perioperative anesthesia care. In our study, based on a retrospective analysis, the utilization of esketamine was identified as an independent factor influencing PD3.

In evaluating patient-related risk factors, diabetes mellitus, BMI are known to have higher risk for morbidity and readmission following all arthroscopy [14]. While our results confirmed that diabetes mellitus or BMI was not an independent influencing factor. Surgical factors, including ligamentous repair, operations involving 3 or more Current Procedural Terminology (CPT) codes are proved to be risk factors for complications after arthroscopic knee surgery [2]. Nevertheless, little is known about the anesthesia-related factors that could facilitate better prognosis for patients.

As an anesthetic and analgesic drug, ketamine is widely used for perioperative pain management [15–18]. Its potent analgesic effect can even last up to 48 h after surgery, significantly reducing the need for postoperative opioid use [19]. Over the past two decades, clinical research has increasingly recognized the clinical benefits that ketamine can bring to patients [20]. In addition to its aforementioned postoperative analgesic effects, it can also reduce the incidence of postoperative nausea and vomiting and improve postoperative depression [21–23]. Esketamine, as compared to conventional ketamine, exhibits a more potent analgesic effect and a faster in vivo clearance rate, despite being administered at only half the dosage [20]. However, few studies have explored whether esketamine can ultimately provide benefits in terms of post-operative hospitalization days. The principal finding of this study was the identification of esketamine use as independent factor influencing PD3.Additionally, ketamine has gained increasing attention for its effective intraoperative anti-inflammatory properties [24]. A previous meta-analysis showed that ketamine significantly reduces the expression of the pro-inflammatory cytokine IL-6 after surgery and increases the expression of the anti-inflammatory cytokine IL-10 [25]. Therefore in future investigations, it would be of value to delve into the potential of esketamine in modulating the inflammatory response profile of knee joint cells that are intricately associated with inflammation, including chondrocytes and synovial cells. However, our investigation did not reveal any significant improvement in postoperative wound infection with the administration of esketamine (Table 5), which may be attributed to inadequate sample size in our study.

A longer surgery duration has previously been identified as a risk factor for extended length of stay, increased transfusion risk, wound dehiscence, death, surgical-site infection, sepsis and hospital readmission in patients underwent knee arthroscopic surgery [2]. Consistent with the previous study, our work demonstrated that an increase in operative time was associated with a decreased ratio of PD3(OR = 0.99, 95% CI(0.97-1.00).Despite the weak correlation between surgery duration and postoperative outcomes observed in our study, the finding highlights the importance of considering surgery duration as a modifiable risk factor in perioperative management, which would have significant implications for optimizing perioperative strategies and improving patient outcomes in clinical practice.

However, limitations of this study include the retrospective nature in which analysis was performed in a single center. There likely exists large variability in patient characteristics prior to knee arthroscopic surgery. Established risk factors influencing hospital stay such as surgery type, operative duration were collected in our study and controlled for multivariate analysis, however postoperative pain score was not recorded, which may lead to unpredictable bias. Furthermore, several clinical studies have tried including the surgical skill level of surgeons in regression models in order to better explain the impact on outcomes. Although this study lacks correction factors for the surgical skill level, the arthroscopic knee surgery was only performed by one fixed surgical team, thus avoiding any confounding effect on outcome analyses. Additionally, it is imperative to acknowledge the existence of variations in perioperative doses of esketamine, as such differences are inherent in retrospective analyses. Recording dosage and the timing of intraoperative analgesics would provide valuable guidance for anesthesiologists in clinical practice. Future studies focused on knee arthroscopic surgery should be more rigorous methodologically.

## Conclusion

The utilization of esketamine may potentially contribute to the reduction of postoperative hospital stay in patients underwent knee arthroscopic surgery. Future studies with a robust methodological approach to validate this result would be of significance for anesthesiologists to optimize the utilization of esketamine.

## Data Availability

The datasets analyzed during the current study are available from the corresponding author upon reasonable request.
